# Ferroptosis in Intervertebral Disc Degeneration: How the Extreme Microenvironment Rewires a Cell Death Network

**DOI:** 10.3390/biomedicines14102397

**Published:** 2026-10-20

**Authors:** Shijiang Geng, Haoran Guo, Shengsheng Tian, Qizhuo Miao, Yang Diao, Xin Wang

**Affiliations:** The 1st School of Clinical Medicine, The First Hospital of Lanzhou University, Lanzhou 730000, China15704897945@163.com (H.G.); tss19991225@163.com (S.T.); miaoqzh2025@lzu.edu.cn (Q.M.); diaoy2024@lzu.edu.cn (Y.D.)

**Keywords:** intervertebral disc degeneration, ferroptosis, Piezo1, ferritinophagy, NCOA4, SLC7A11, Nrf2, microenvironment

## Abstract

Ferroptosis, an iron-dependent regulated cell death driven by lipid peroxidation, contributes to intervertebral disc degeneration (IVDD), yet most reviews describe its pathways without asking why the disc—the largest avascular tissue, combining hypoxia, nutrient deprivation, mechanical loading, acidosis, and low-grade inflammation—regulates ferroptosis differently from other tissues. Here we address this question along three axes. First, the execution machinery shows three IVDD-specific vulnerabilities: a proposed diffusion-limited cystine “semi-starvation”, mechanically induced GPX4 inhibition via the Piezo1–Ca^2+^ axis, and epigenetic GCLC suppression. Second, iron homeostasis undergoes a “triangular imbalance”: TFR1 upregulation coexists with restricted iron supply, acidosis-activated NCOA4 ferritinophagy is proposed as the dominant source of labile iron (with the UCHL3–HMGB1–NCOA4 axis as a newly identified upstream regulator), and FPN1 export is blocked by the IL-6–hepcidin axis, predisposing Nrf2–HO-1 to a protective-to-deleterious functional reversal. Third, inflammation and ferroptosis form a vicious cycle in which STAT3 signals through the non-canonical S100A6–p53 axis—opposite to its tumour-protective FTH1 role—jointly imposing a “dual lock” on SLC7A11. Integrating these findings, we construct a network in which Nrf2 is proposed as the transcriptional hub and SLC7A11 as the convergent bottleneck, and derive an SLC7A11-restoration hypothesis predicting that multi-target combinations, not single-pathway interventions, will be required—a prediction that remains to be tested. We close by discussing the controversies and a prioritized research agenda.

## 1. Introduction

### 1.1. Intervertebral Disc Degeneration: A Disease Defined by Its Microenvironment

Intervertebral disc degeneration (IVDD) is the most common cause of low back pain and cervical radiculopathy, affecting approximately 266 million people worldwide, with a prevalence of ~40% in individuals under 40 and over 90% in those above 50 [1]. The intervertebral disc is the largest avascular tissue in the human body [2,3]: oxygen tension in its central region is as low as 1–5%, glucose concentration is merely 10–20% of plasma levels, and nutrition relies entirely on diffusion through the cartilaginous endplates [4,5,6]. This anatomical singularity fundamentally distinguishes IVDD from other degenerative diseases such as osteoarthritis and neurodegeneration. As degeneration progresses, endplate calcification further impairs permeability, while aberrant mechanical loading and low-grade inflammation act continuously on the surviving nucleus pulposus (NP) cells. Avascularity, hypoxia, mechanical overload, acidosis, and low-grade inflammation therefore jointly define the stage on which every cellular behaviour in IVDD unfolds; any mechanistic discussion of IVDD must be examined against these five microenvironmental elements.

### 1.2. Ferroptosis: A New Perspective on Cell Death in IVDD

Ferroptosis, named in 2012, is an iron-dependent, non-apoptotic form of cell death characterized by impaired GPX4-mediated lipid peroxide detoxification and accumulation of lipid peroxides that ultimately disrupt membrane integrity [7,8,9]. Since 2018, evidence has accumulated for iron accumulation, decreased GPX4 activity, and elevated lipid peroxidation products (e.g., 4-HNE) in degenerative NP tissue [10,11,12], establishing ferroptosis as an important mode of NP cell death in IVDD [10,11,12]. The pathogenic relevance of ferroptosis extends across degenerative diseases: it drives progressive neuronal loss in neurodegenerative disorders [13] and disrupts chondrocyte homeostasis in osteoarthritis [14], underscoring the general importance of this pathway in tissue degeneration. However, most published studies remain at the correlational level—detecting ferroptosis markers and inferring involvement—without systematically analyzing why ferroptosis regulation adopts tissue-specific patterns in the disc [11,12,15,16].

### 1.3. Scope and Core Question of This Review

This review addresses one core question: how does the extreme microenvironment of IVDD specifically rewire the ferroptosis regulatory network? The main text proceeds from the execution machinery to iron metabolism, inflammatory crosstalk, and finally the integrated network: Section 2 analyzes the three vulnerabilities of the ferroptosis execution machinery (the GSH–GPX4 axis) in IVDD; Section 3 dissects the “triangular imbalance” of iron metabolism and its unique origins in an avascular tissue; Section 4 elaborates the inflammation–ferroptosis vicious cycle, emphasizing the non-canonical pro-ferroptotic axis of STAT3; Section 5 constructs the integrated regulatory network centred on the Nrf2 hub and the SLC7A11 bottleneck; Section 6 derives therapeutic implications; and Section 7 concludes with controversies and future directions. Table 1 maps the microenvironment-to-mechanism relationships that constitute the backbone of this review.

**Table 1:** Mapping of IVDD microenvironmental features to ferroptosis perturbations.

Microenvironmental Feature	Molecular Mediator	Ferroptosis Node Affected	Consequence	Evidence Strength	Section
Avascularity/nutrient deprivation	Endplate calcification → reduced permeability	Trans-endplate cystine transport	Cystine “semi-starvation” (proposed) compounding SLC7A11 transcriptional suppression	Indirect (physical models + imaging; direct intradiscal measurements lacking)	Section 2.1
Aberrant mechanical loading	Piezo1 overactivation → pathological Ca^2+^ influx	GPX4 activity	Mechanically induced GPX4 inhibition downstream of Piezo1–Ca^2+^ signalling; selenoproteins (SelK)as natural counterbalance	Moderate (in vitro + animal models)	Section 2.2
Acidic metabolism (pH ≤ 6.5)	Lactate accumulation	NCOA4-mediated ferritinophagy	Stored iron release; regulated by the UCHL3–HMGB1 axis and RBX1-mediated ubiquitination	Moderate (2025–2026 evidence)	Section 3.2
Low-grade inflammation	NF-κB/STAT3	SLC7A11 transcription	“Dual lock” on the common bottleneck via direct and p53-relayed suppression	Moderate (STAT3 axis); Indirect (NF-κB–SLC7A11 repression)	Section 4.1, Section 4.2 and Section 4.3
Hypoxia	Constitutive HIF-1α (attenuating Nrf2-dependent transcription)	TFR1 expression/Nrf2 nuclear translocation	Iron import upregulation coexisting with impaired Nrf2 activation	Moderate (TFR1 upregulation); Indirect (HIF-1α–Nrf2 crosstalk)	Section 3.1 and Section 5
Oxidative stress	Sustained ROS production	GCLC expression (miR-27a/promoter methylation)	A new rate-limiting step at the GSH synthesis end	Hypothetical (awaiting direct validation in NP cells)	Section 2.3

**Figure 1:**
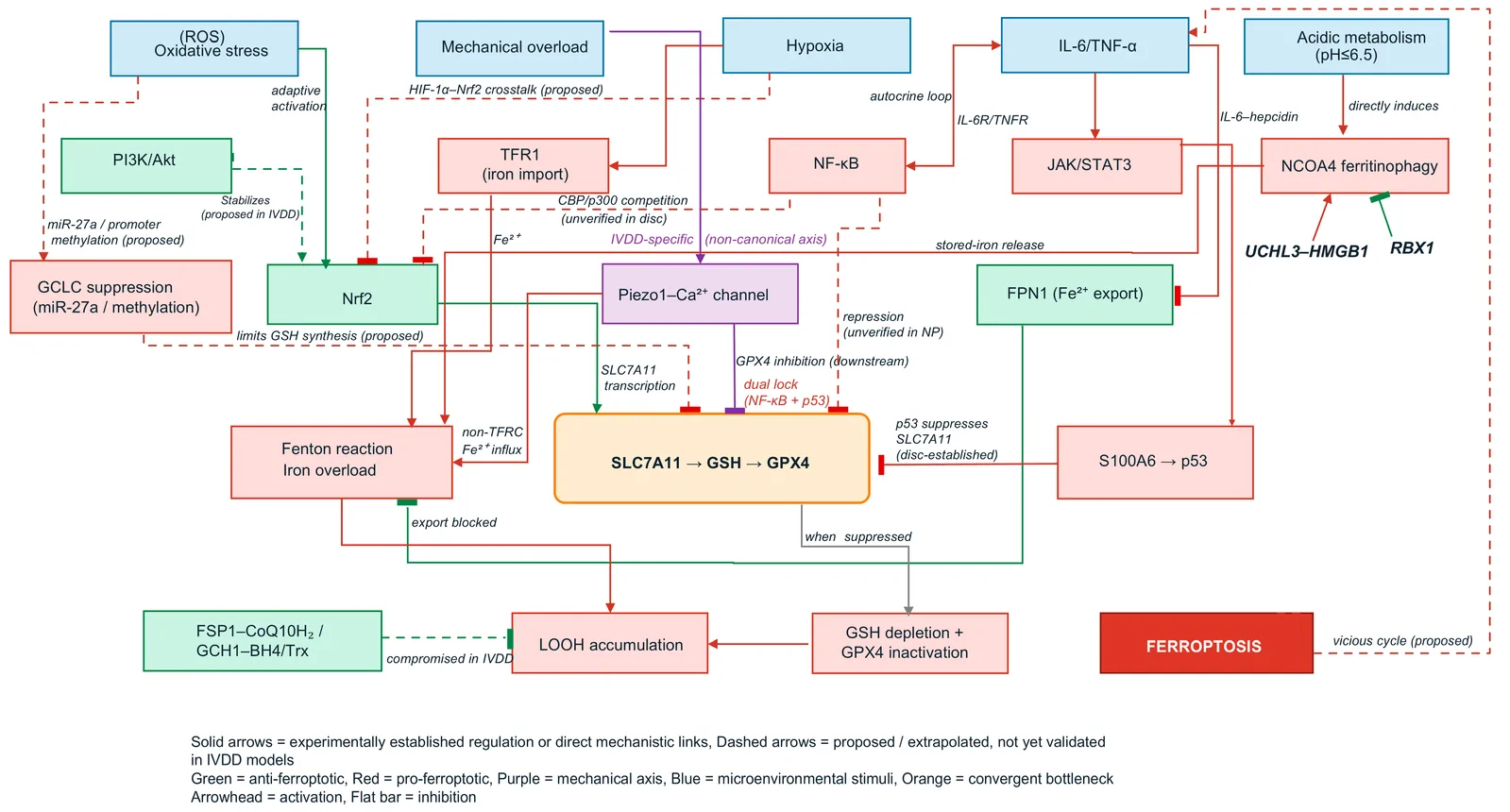
Multiple signalling axes converge on the common bottleneck SLC7A11–GSH–GPX4 axis to regulate ferroptosis in IVDD. Anti-ferroptotic signals (Nrf2) promote SLC7A11; mechanical, iron and inflammatory axes suppress GPX4 or perturb iron homeostasis, driving lipid peroxidation and ferroptosis. Solid arrows denote experimentally established transcriptional regulation or direct mechanistic links; dashed arrows denote proposed or extrapolated interactions not yet validated in IVDD models.

Each microenvironmental element exerts pressure on a distinct node of the ferroptosis defence, with convergent amplification on the SLC7A11–GSH–GPX4 axis (Figure 1).

### 1.4. Search Strategy and Evidence Grading

Literature was retrieved from PubMed, Web of Science, and Scopus up to August 2026 using combinations of the terms “ferroptosis”, “intervertebral disc degeneration”, “nucleus pulposus”, “SLC7A11”, “GPX4”, “NCOA4”, “ferritinophagy”, “Piezo1”, “hepcidin”, “NF-κB”, and “STAT3”, without language restriction; reference lists of retrieved reviews were screened for additional records. Given the narrative nature of this review, no PRISMA-based screening was performed. Instead, every central claim is graded according to a unified four-level evidence scheme based on the experimental model supporting it: Strong (reproduced human tissue evidence or converging independent cell and animal evidence in IVDD), Moderate (cell or animal evidence in IVDD, typically from a single group), Indirect (evidence transferred from other tissues or disease models, cited by analogy and stated as such), and Hypothetical (untested proposals awaiting direct validation in nucleus pulposus cells). This grading is applied throughout the text, summarized in the Evidence Strength column of Table 1, Table 2 and Table 3 and Supplementary Table S1, and encoded in Figure 1, where solid arrows denote experimentally established regulation and dashed arrows denote proposed or extrapolated interactions.

## 2. The Ferroptosis Execution Machinery in IVDD: Three Vulnerabilities

The ferroptosis execution machinery comprises three modules: cystine is imported via System Xc^−^ (SLC7A11/SLC3A2heterodimer) [54,55], GSH is synthesized from cysteine through the GCL- and GS-catalyzed two-step reaction [56], and GSH serves as the cofactor enabling GPX4 to reduce toxic lipid hydroperoxides (LOOHs) to non-toxic lipid alcohols (LOH). In most tissues, the weak point of this defence resides in the transcriptional regulation of SLC7A11 [57]. In the intervertebral disc, however, the microenvironment imposes pressure at three additional, IVDD-specific sites.

To appreciate these disc-specific vulnerabilities, it is useful to first outline the opposing pro-ferroptotic arm of the system. Intracellular free iron (Fe^2+^) catalyzes the Fenton reaction, generating hydroxyl radicals that attack polyunsaturated fatty acid (PUFA)-containing phospholipids [9]; ACSL4- and LPCAT3-mediated esterification primes membrane phospholipids for peroxidation, which LOX enzymes then convert into LOOH [7,24]. Unless detoxified, LOOH propagates radical chain reactions within membranes and decomposes into reactive aldehydes such as 4-hydroxynonenal (4-HNE), culminating in membrane rupture [24]. The anti-ferroptotic side comprises at least two parallel layers: the canonical GSH–GPX4 axis described above and the GSH-independent FSP1–CoQ10H_2_ radical-trapping system [24]. In IVDD, the esterification arm is pharmacologically tractable: Duhuo Jisheng Decoction suppresses NP cell ferroptosis through exosomal miR-19b-3p targeting of ACSL4 [47], providing functional evidence that this arm operates in the disc—although whether ACSL4 expression itself is upregulated in human degenerated tissue remains to be quantified. In healthy NP cells, both layers operate with adequate capacity; degeneration erodes them at tissue-specific points, as detailed below.

### 2.1. First Vulnerability: Cystine “Semi-Starvation” in an Avascular Tissue

NP cells depend entirely on diffusion through the cartilaginous endplates for nutrient supply [3,4]. During degeneration, endplate calcification progressively reduces permeability, lowering the trans-endplate transport efficiency of cystine and other small-molecule substrates. Compared with well-vascularized tissues, degenerative NP cells may exist in a state of cystine “semi-starvation”, in which GSH synthesis substrates become limiting; this state is proposed on the basis of physical models of nutrient transport and imaging evidence of endplate calcification, and direct measurement of cystine concentrations in degenerative disc interstitial fluid remains an outstanding experiment. If confirmed, superimposed transcriptional suppression of SLC7A11 (Section 4 and Section 5) would create a dual insult at both the substrate and transporter ends.

### 2.2. Second Vulnerability: Mechanical Loading Inhibits GPX4 via the Piezo1–Ca^2+^ Axis

Another defining feature distinguishing IVDD from other degenerative diseases is sustained aberrant mechanical loading [58]. Recent studies have delineated the pathway linking mechanical stimuli directly to the ferroptosis execution machinery: under excessive compressive load, the mechanosensitive ion channel Piezo1 is pathologically activated in NP cells, inducing Ca^2+^ influx that mediates non-TFRC-dependent iron overload; this channel-mediated iron influx, together with a concurrent decline in GPX4 activity (an indirect downstream consequence rather than a direct action of the channel on the enzyme), drives lipid peroxidation and ferroptosis [22,59]. This Piezo1–Ca^2+^–ferroptosis axis establishes, at the molecular level, that mechanical loading is not merely a background factor of degeneration but a direct trigger of ferroptosis.

Notably, the selenoprotein pathway provides a natural counterbalance to this mechanical axis: selenium supplementation upregulates selenoproteins such as SelK, enhancing GPX4 activity and antagonizing Piezo1-mediated ferroptosis, thereby alleviating oxidative stress and disc senescence [36]. This suggests that the balance between mechanical load intensity and selenium nutritional status may determine whether NP cells survive or undergo ferroptosis under mechanical stress—a testable framework for understanding inter-individual variability in IVDD. The selenium dimension deserves particular emphasis because GPX4 itself is a selenoprotein: its translation requires the SECIS element and selenium-dependent insertion of selenocysteine, so selenium deficiency directly impairs GPX4 synthesis at the translational level [24]. In the nutritionally deprived disc—where cystine supply is proposed to be diffusion-limited (Section 2.1)—selenium availability may constitute a second, parallel nutrient bottleneck on the very same defence enzyme. Whether trans-endplate selenium flux is sufficient in the degenerating disc, and whether selenium status correlates with IVDD severity in patients, are both untested and represent an open epidemiological question amenable to relatively low-cost clinical studies.

### 2.3. Third Vulnerability: Epigenetic Suppression of GCLC—A New Rate-Limiting Step in GSH Synthesis

GCL, the rate-limiting enzyme of GSH synthesis, comprises a catalytic subunit (GCLC) and a modulatory subunit (GCLM) [60,61]. In degenerative NP cells, GCLC expression may be suppressed at the epigenetic level through two mechanisms: hypermethylation of its promoter and upregulation of miR-27a, which targets the GCLC mRNA 3′UTR. Direct experimental evidence for the miR-27a mechanism currently exists only outside the disc: miR-27a-3p suppresses GCLC-dependent glutathione biosynthesis in oxidative-stress models [62], and miR-27a is expressed in NP cells in an autophagy-related context [63]. If confirmed in NP cells, this would imply that GSH depletion in IVDD is not only a problem of consumption (GPX4 inactivation, excessive ROS) but also of production (GCLC transcriptional repression). The therapeutic corollary is important: merely enhancing cystine uptake through Nrf2-mediated SLC7A11 activation may be insufficient to restore GSH levels, because GCLC repression would constitute a new rate-limiting bottleneck requiring epigenetic intervention or alternative GSH precursors. This hypothesis currently lacks direct validation in degenerative NP cells and represents a high-priority experimental direction.

### 2.4. The Second Defence Layer: Compensatory Pathways Under the Same Microenvironmental Pressure

Because the GSH-dependent layer is systematically eroded in IVDD, the fate of NP cells also depends on the GSH-independent defence layer shown in Figure 1. The FSP1–CoQ10H_2_ system traps lipid radicals independently of GSH [24], yet its substrates are vulnerable to the same microenvironmental constraints that weaken the canonical axis: FSP1 requires NAD(P)H, whose availability may be compromised by the mitochondrial dysfunction and hypoxia of the degenerating disc—a proposal that currently lacks direct intradiscal measurements—and CoQ10synthesis depends on mevalonate-pathway intermediates whose supply in the nutritionally deprived NP cell is unverified. Consistent with these constraints, multi-omics profiling of degenerated discs identified reduced FSP1 alongside depressed GPX4 [26], suggesting that the compensatory layer fails in parallel rather than compensating—an important point, because Nrf2-centric interventions that restore the GSH–GPX4 axis may leave the disc dependent on a second layer that is itself compromised. The GCH1–BH4 and Trx–TrxR systems, which counter ferroptosis by inhibiting LOX activity and scavenging ROS, respectively, have not been examined in IVDD at all, leaving their contribution entirely unknown. Finally, the mitochondrial DHODH–CoQH_2_ pathway represents a third, inner-membrane defence that operates in parallel to GPX4; in the disc, it is known only as a target activated by the DFOM@CeO_2_ nanoformulation [30], with no direct evidence for its endogenous regulation in NP cells. These gaps define a clear experimental agenda for the compensatory layer in IVDD.

## 3. The “Triangular Imbalance” of Iron Metabolism: Collapse of Iron Homeostasis in an Avascular Tissue

Iron dysmetabolism is the initiating condition for ferroptosis. Normal NP cells maintain an “iron-thrifty” phenotype—low TFR1 expression, high FPN1 expression, and high ferritin storage—adapting to extremely limited iron availability. During degeneration, iron import, stored-iron release, and iron export destabilize simultaneously, forming a characteristic “triangular imbalance”.

### 3.1. A Paradox: TFR1 Upregulation Amid Restricted Iron Supply

TFR1 upregulation in degenerative NP tissue is consistently observed, consistent with iron-import programmes driven by NF-κB and HIF signalling documented in other systems [22,64]. Yet this finding embeds a paradox that has received little attention: given the reduced permeability caused by endplate calcification, whether transferrin-bound iron concentrations in the disc interstitial fluid suffice to support increased TFR1-mediated iron influx is unverified. Some investigators have therefore proposed that the contribution of TFR1 upregulation may be overestimated and that ferritinophagy-mediated release of stored iron is the more important source of labile iron [20].

### 3.2. NCOA4-Mediated Ferritinophagy: The True Iron Source Driven by the Acidic Microenvironment

Ferritinophagy is the selective autophagic degradation of ferritin via the cargo receptor NCOA4, releasing stored iron. Multiple lines of evidence place it at the core of iron overload in IVDD. First, a 2025 study showed that the acidic microenvironment of the degenerative disc (pH ≤ 6.5, resulting from lactate accumulation) directly induces NCOA4-mediated ferritinophagy and ferroptosis in NP cells; reduced serum ferritin is an independent risk factor for IVDD; and the E3 ubiquitin ligase RBX1 suppresses ferritinophagy by ubiquitinating NCOA4, with RBX1 overexpression alleviating IVDD in vivo [20]. This anchors the trigger of ferritinophagy directly to microenvironmental acidity [65]—a hallmark feature of IVDD and a key tissue specificity of the disc.

Second, a newly reported upstream regulatory axis further enriches this picture: the deubiquitinase UCHL3, upregulated in degenerative discs, removes Lys48-linked polyubiquitin chains from HMGB1, stabilizing it and promoting its cytoplasmic accumulation, which in turn activates NCOA4-mediated ferritinophagy and drives NP cell ferroptosis; silencing UCHL3 or NCOA4 mitigates these effects [21]. The UCHL3–HMGB1–NCOA4 axis extends the regulatory hierarchy of ferritinophagy from execution to post-translational modification, offering new intervention sites [26,31].

Mechanistically, NCOA4 delivers ferritin to autophagosomes through interaction with LC3, and lysosomal fusion releases iron in a pH-dependent manner [20,24]; in NP cells, this process is activated in a ULK1 phosphorylation-dependent manner [20]. These findings explain why ferritinophagy-derived iron, rather than transferrin-bound import, is proposed to dominate the labile iron pool in advanced degeneration.

In summary, in IVDD, release of stored iron rather than extracellular iron import is likely the primary driver of labile iron pool expansion—in stark contrast to iron-overload diseases such as hemochromatosis, which are dominated by excessive iron absorption. This also explains why TFR1-blocking interventions alone may have limited efficacy.

### 3.3. FPN1 Blockade: The IL-6–Hepcidin Axis Closes the Loop

At the iron export side, FPN1 is the only known cellular iron exporter [66]. Hepcidin binds FPN1 and induces its internalization and degradation [67]. This axis has now received direct experimental support in IVDD: HIF-2α activates IL-6/hepcidin/FPN1 signalling to promote NP cell ferroptosis and aggravate disc degeneration [25], and FPN1-dependent iron export protects NP cells against oxidative stress-induced ferroptosis in vivo [68]. Together with persistent IL-6 expression in the inflammatory microenvironment, these findings establish the export blockade as an experimentally supported component—not merely a theoretical one—of the “one-way iron trap”. The simultaneous destabilization of iron import (TFR1↑), stored-iron release (NCOA4↑), and iron export (FPN1↓) constitutes the “triangular imbalance” framework of iron dysmetabolism in IVDD [20].

### 3.4. The “Protective-to-Deleterious” Functional Reversal of Nrf2–HO-1: An Underappreciated Transition

HO-1 catalyzes heme degradation into biliverdin, CO, and Fe^2+^. The first two are antioxidant and anti-inflammatory mediators, whereas Fe^2+^ directly expands the labile iron pool. In normal NP cells, FPN1 exports HO-1-derived Fe^2+^ in a timely manner, keeping the net effect protective; in degenerative NP cells, however, FPN1 is suppressed by hepcidin (Section 3.3), and HO-1-generated Fe^2+^ can no longer be exported, tipping the balance between the protective and pro-death faces. The literature has explicitly recognized the dual nature of HO-1 in ferroptosis: excessive activation releases iron beyond the neutralizing capacity of ferritin while consuming NADPH, expanding the labile iron pool and promoting ferroptosis [23]. In the disc, HO-1 is generally cytoprotective in NP-derived cells [69], yet BACH1—a transcriptional repressor of HMOX1—promotes NP cell ferroptosis via the HMOX1/GPX4 axis, whereas BACH1 deletion activates Nrf2/HO-1 and alleviates ferroptosis [70]. Therefore, in the FPN1-suppressed degenerative microenvironment, the Nrf2–HO-1 axis may undergo a “protective-to-deleterious” functional reversal [24].

The Nrf2–BACH1 axis further illustrates this dosage dependence. BACH1 competes with Nrf2 for antioxidant response elements and constitutively represses HMOX1; its activity is restrained by heme-induced degradation under basal conditions. In NP cells, BACH1 upregulation promotes ferroptosis through HMOX1/GPX4 dysregulation, whereas BACH1 deletion relieves this repression and activates Nrf2/HO-1-mediated protection [70]. This dual regulation implies that HO-1 output in the degenerating disc reflects not merely the abundance of activators but the balance between Nrf2-mediated induction and BACH1-mediated repression—and that therapeutic strategies aimed at HO-1 must consider which side of this balance they modulate.

This reversal hypothesis carries important therapeutic implications: in advanced degeneration (FPN1 already suppressed), potent HO-1 inducers may be not only ineffective but harmful. Identifying the time window of this reversal and its biomarkers is a prerequisite for determining when and what to intervene.

## 4. The Inflammation–Ferroptosis Vicious Cycle: A Hallmark of IVDD

The degenerative disc harbours a persistent low-grade inflammatory microenvironment. NP cells, stimulated by aberrant mechanics and nutrient deprivation, actively secrete IL-6 and TNF-α, which drive ferroptosis through the NF-κB and JAK/STAT3 axes, establishing a feed-forward loop of “inflammation → ferroptosis → further cell death → further cytokine release”. Direct evidence for this vicious cycle in IVDD comes from polydopamine-based iron-chelating nanoparticles that interrupt the ROS–ferroptosis–inflammation loop and alleviate degeneration [27]. This loop is hypothesized to mark the transition from reversible functional alterations to irreversible structural destruction in IVDD [71].

### 4.1. NF-κB: Initiator of the Inflammation–Ferroptosis Vicious Cycle

Activated NF-κB exerts pro-ferroptotic effects through three mechanisms, whose evidence levels in the disc differ substantially. (1) Under sustained inflammatory stimulation, NF-κB can transcriptionally repress SLC7A11 and thereby restrict GSH synthesis substrates [72,73]; however, this regulation is context-dependent—in several systems NF-κB instead activates SLC7A11—and has not been directly validated in nucleus pulposus cells. (2) NF-κB signalling is associated with upregulated TFR1 and LOX (particularly ALOX15) expression, strengthening iron import and the enzymatic basis of PUFA peroxidation; this is supported by TNF-α/NF-κB–TFRC evidence in other systems [64], whereas only indirect phenomena have been reported in the disc, without promoter-level (ChIP) evidence. (3) NF-κB promotes TNF-α and IL-6 transcription and secretion, forming an autocrine/paracrine positive feedback loop [27]; in NP cells, this loop is sustained by oxidized low-density lipoprotein through a LOX-1/NF-κB/NOX feed-forward circuit [74]. In addition, NF-κB p65 competes with Nrf2 for the transcriptional coactivator CBP/p300—a classic mechanism established in inflammatory models [75,76]—indirectly suppressing Nrf2 downstream antioxidant genes; whether this competition operates in disc cells remains unverified (Figure 1).

### 4.2. The Non-Canonical Pro-Ferroptotic Axis of STAT3: The Most Distinctive Finding in IVDD

**Figure 2:**
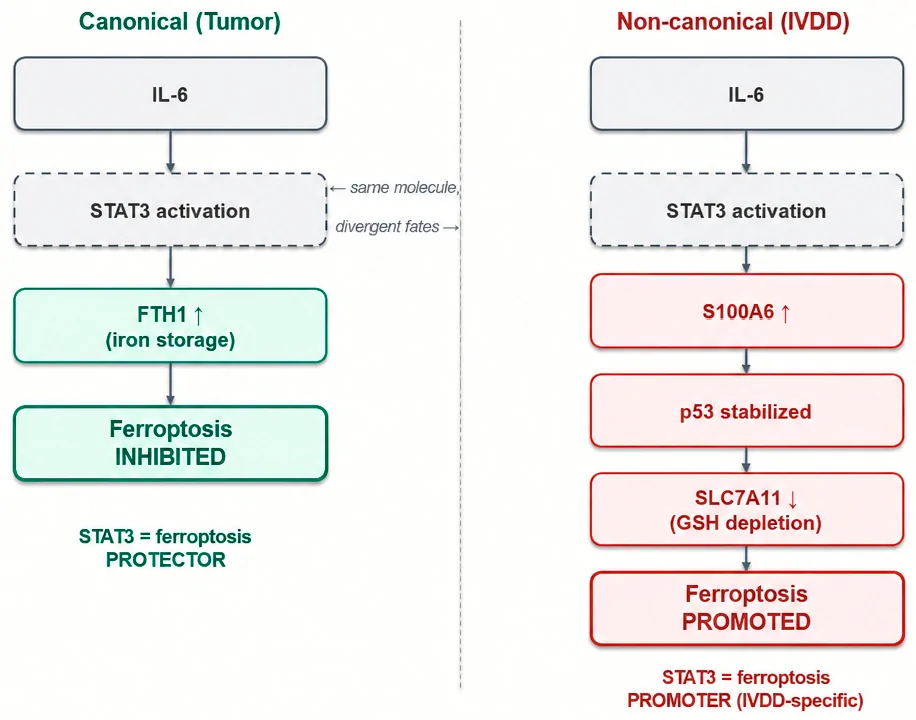
In tumours, STAT3 transcriptionally activates FTH1 to sequester iron and inhibit ferroptosis; in the degenerating nucleus pulposus, STAT3 instead stabilizes p53 via S100A6, suppressing SLC7A11 and promoting ferroptosis.

The role of STAT3 in ferroptosis regulation is highly context-dependent. In most tumour models, STAT3 translocates to the nucleus and directly transcribes FTH1, increasing iron storage and thereby inhibiting ferroptosis—STAT3 is viewed as a ferroptosis “protector” [17,18]. In degenerative NP cells, however, a 2025 study revealed a strikingly opposite mode [19]: IL-6/JAK-activated STAT3 does not upregulate FTH1; instead, it binds the S100A6 promoter to upregulate its expression; S100A6, a Ca^2+^-binding protein, interacts with the C-terminus of p53, stabilizing p53 and promoting its Ser15 phosphorylation and nuclear translocation; nuclear p53 then suppresses SLC7A11 transcription, ultimately promoting ferroptosis through GSH depletion and GPX4 inactivation. Thus, STAT3 in IVDD operates through the non-canonical “STAT3→S100A6→p53→SLC7A11↓” pro-ferroptotic pathway, rather than the canonical “STAT3→FTH1↑” protective pathway observed in tumours (Figure 2).

Why STAT3 abandons the FTH1 pathway in IVDD remains incompletely understood. Three possibilities are proposed: (1) the hypoxic microenvironment—constitutive HIF-1α expression may hinder STAT3 binding to the FTH1 promoter through protein–protein interactions; (2) epigenetic alterations—FTH1 promoter hypermethylation or repressive histone modifications may render this locus inaccessible; and (3) the S100A6–p53 axis may competitively “redirect” STAT3 transcriptional activity toward pro-ferroptotic gene networks. Furthermore, multiple upstream routes can activate STAT3 in the disc: IL-10 from M2-polarized macrophages [77], periostin (POSTN) [78], and endoplasmic reticulum stress acting through PERK [79,80]—yet whether different upstream stimuli produce “STAT3 downstream effector bias” remains entirely unexplored. The identification of this non-canonical axis is the most distinctive finding in the IVDD ferroptosis field and the strongest evidence for this review’s central thesis of microenvironment-driven network rewiring.

Beyond SLC7A11 suppression, p53 exerts additional pro-ferroptotic activities that amplify its position as the effector of the STAT3 relay: it promotes ferroptosis by driving DPP4 plasma-membrane localization, by directly repressing GPX4 transcription, and by inducing FDXR-mediated iron metabolism genes [18,24]. None of these mechanisms has been examined in NP cells, but their convergence on the same downstream machinery suggests that p53 activation in the degenerating disc—whether from senescence or from the STAT3–S100A6 relay—could strike the ferroptosis defence through multiple, partially redundant routes.

### 4.3. Crosstalk and the “Dual Lock” on SLC7A11

Crosstalk between NF-κB and STAT3 integrates the two pathways into network-level behaviour: IL-6 released upon NF-κB activation is among the most potent upstream activators of STAT3 [18,77]—NF-κB not only promotes ferroptosis directly but also amplifies pro-ferroptotic signalling through the IL-6–JAK–STAT3 axis. Simultaneously, p53 downstream of STAT3 further suppresses SLC7A11 [19], forming a “dual lock” on SLC7A11 together with NF-κB’s context-dependent transcriptional repression—two pathways converging on the same target gene through distinct mechanisms (Figure 1). A direct corollary of this convergent architecture is that activating Nrf2 alone to upregulate SLC7A11 may be insufficient to overcome the combined repression exerted by NF-κB and p53; combined “Nrf2 activation + NF-κB/STAT3 inhibition” is predicted to be necessary, although this prediction has not yet been tested experimentally (Section 6).

### 4.4. Ferroptosis Amid Other Cell Death Modalities in the Disc

Ferroptosis does not operate in isolation: apoptosis, pyroptosis, and necroptosis coexist in the degenerating disc, and the regulatory machinery described above intersects them at shared nodes. p53 simultaneously governs apoptotic and ferroptotic programmes, so the STAT3–S100A6–p53 relay (Section 4.2) may, in principle, drive both outcomes depending on context; NF-κB orchestrates pyroptosis priming while sustaining the inflammatory-ferroptotic loop described in Section 4.1; and RIPK1/RIPK3 effectors are modulated by the same oxidative environment that fuels lipid peroxidation [11]. Two implications follow. First, whether NP cells die by ferroptosis or by an alternative modality under a given microenvironmental insult may depend on the relative kinetics of competing pathways rather than on a single decisive signal—an unresolved question with direct consequences for interpreting single-modality interventions. Second, pharmacological inhibition of one modality may divert cells toward another, a possibility that could explain the partial in vivo efficacy of ferroptosis-only strategies and that argues for combined analyses of death markers in future studies.

## 5. Convergent Regulation: The Nrf2 Hub and the SLC7A11 Bottleneck

Before synthesizing the network, two upstream regulatory inputs depicted in Figure 1 require textual anchoring. First, PI3K/Akt is the principal endogenous anti-ferroptotic signal in the disc, acting through two parallel branches: it stabilizes Nrf2 activity and sustains SREBP1/SCD1-mediated monounsaturated fatty acid synthesis, which maintains a membrane lipid composition resistant to peroxidation. Its relevance is currently supported mainly by therapeutic evidence—Duhuo Jisheng Decoction and ginsenoside Rg3 alleviate IVDD at least partly through PI3K/AKT activation [48,51]—whereas whether endogenous PI3K/Akt activity progressively declines during degeneration, as proposed for other degenerative diseases, remains to be tested directly in NP cells. Second, the hypoxic environment couples HIF-1α to the same regulatory core: HIF-1α activation attenuates Nrf2-dependent transcription [81], although the underlying molecular mechanism remains incompletely defined; sustained HIF-1α expression could therefore indirectly limit Nrf2 transcriptional output—a possibility that would complement the impaired Nrf2 nuclear translocation noted above, although it has not been examined in disc cells.

Integrating the analyses in Section 2, Section 3 and Section 4, ferroptosis regulation in IVDD exhibits three network-level features that distinguish it from other degenerative diseases (Figure 1; Table 1 and Table 2 for structured summaries).

First, microenvironment-drivenmulti-pathway synergistic inactivation. Avascularity(proposed cystine semi-starvation), mechanical overload (Piezo1–Ca^2+^-mediated GPX4 inhibition), low-grade inflammation (NF-κB/STAT3 axes), and acidic metabolism (pH ≤ 6.5-activated ferritinophagy)weaken the ferroptosis defence at distinct nodes, with synergistic amplification on the SLC7A11–GSH–GPX4 axis. The expected benefit of single-pathway interventions is therefore systematically overestimated: a Nrf2 activator alone cannot compensate for the substrate deprivation imposed by endplate calcification, nor does it blunt Piezo1-mediated GPX4 inhibition.

Second, SLC7A11 is the common bottleneck. Regardless of whether upstream pressure originates from NF-κB (inflammation), p53 (senescence/STAT3 relay), or impaired Nrf2 nuclear translocation (oxidative stress), the ultimate effect converges on the common pathway of SLC7A11 transcriptional suppression → GSH depletion → GPX4 inactivation. Substrate insufficiency (avascularity) acts at a different node: it does not regulate SLC7A11 transcription but limits the substrate pool available to the transporter, so that even intact transporter function cannot fully compensate. Transporter regulation and substrate limitation therefore constitute distinct bottlenecks that converge only at the level of GSH synthesis. This convergent architecture also rationalizes why single-agent TFR1 blockade performs poorly: iron continues to accumulate through ferritinophagy, and GPX4 remains repressed by the dual lock. This bottleneck position makes SLC7A11 the most attractive single target but also means it bears the combined suppression of multiple pathways.

Third, “protective-to-deleterious” functional reversals are widespread. Pathways such as Nrf2–HO-1 (Section 3.4) and AMPK–mitophagy (protective when moderate, harmful when excessive) may reverse their net effect during the course of degeneration. Similarly, mitophagy—protective in early degeneration—becomes destructive when mitochondrial mass falls below a critical threshold, echoing the time-window problem. Identifying the time windows and biomarkers of these reversals is the central prerequisite for defining the therapeutic window.

An important caveat qualifies the “hub” designation assigned to Nrf2 above. Direct causal evidence for Nrf2 in IVDD ferroptosis—through conditional knockout or overexpression in disc models—is essentially absent; current support derives mainly from pharmacological activators (cynarin, fisetin, Rg1, nystose; Table 3), whose effects are consistent with, but do not prove, a central endogenous role for Nrf2. Moreover, the impaired nuclear translocation observed in degenerative NP cells (Section 3.4 context) suggests that endogenous Nrf2 activity is already compromised in degeneration—a state that pharmacological activation must overcome before upregulating its targets. The hub position of Nrf2 in our network (Figure 1) is therefore a synthesis of convergent but indirect evidence, and validating it causally is a prerequisite for the SLC7A11-restoration strategy derived from it.

Based on these network features, we propose a testable core hypothesis—the “SLC7A11-restoration hypothesis”: the efficacy of any intervention depends on whether it can restore SLC7A11 expression to the threshold level required to withstand the combined repression from multiple inhibitory pathways; activation of a single upstream pathway (e.g., Nrf2 alone) is predicted to be insufficient to reach this threshold, whereas multi-target combinations (Nrf2 activation + NF-κB/STAT3 inhibition + iron chelation) are predicted to produce synergistic efficacy; the predicted necessity, superiority, and synergy of combinations remain untested.

## 6. Therapeutic Implications: From Mechanism to Strategy

Ferroptosis-targeting strategies can be classified by their intervention node into four groups: iron metabolism modulators (DFO, Ferristatin II, DFOM@CeO_2_), lipid peroxidation inhibitors (Fer-1, Lip-1, Resveratrol [82], Quercetin), cell defence and signalling pathway modulators (Nrf2 activators including cynarin, fisetin, and ginsenoside Rg1; epigenetic modifiers including gallic acid derivatives; the STAT3/NFKB1 inhibitor BRD-K20733377), and endogenous signalling peptides (Humanin). A systematic evaluation of targets, experimental models, outcome measures, advantages, limitations, and research stages for these agents is provided in Table 3. As of early 2026, all strategies remain preclinical, with no randomized controlled trials for the IVDD indication.

The mechanistic analysis in this review yields three explicit strategy-level inferences. First, targeting stored-iron release (NCOA4/UCHL3) may offer advantages over targeting iron import (TFR1), because ferritinophagy, rather than import, is hypothesized to be the primary source of labile iron in IVDD (Section 3.2). Second, the efficacy ceiling of Nrf2 monotherapy is likely set by the “dual lock” on SLC7A11 (Section 4.3); combination strategies of “Nrf2 activation + NF-κB/STAT3 inhibition” or “Nrf2 activation + iron chelation” merit priority screening. Third, HO-1-inducing interventions require caution regarding the “protective-to-deleterious” reversal (Section 3.4)—timing and dosage may matter more than drug class.

### Delivery Systems: The Second Bottleneck Independent of Mechanism

Drug delivery constitutes a second bottleneck independent of mechanism. The avascular, hyperosmolar environment of the disc prevents effective systemic drug concentrations, and the dense, negatively charged extracellular matrix impedes the diffusion of charged macromolecules [83,84]. Three delivery paradigms are currently being explored. First, stimuli-responsive hydrogels exploit the pathological signals of the degenerating disc themselves as release triggers: pH-responsive systems capitalize on the acidic microenvironment (pH ≤ 6.5) that also drives ferritinophagy, releasing cargo preferentially at sites of active degeneration; ROS-responsive systems use thioketal or boronic ester linkers that cleave under oxidative stress, coupling drug release to the very ferroptotic burden they aim to suppress. Second, injectable hydrogels provide mechanical support while serving as local drug depots, addressing both the biomechanical and pharmacological needs of the disc in a single intervention. Third, functionalized nanocarriers achieve subcellular targeting: mitochondrial-targeting peptides (e.g., the SS08 peptide in PGA-Cu@SS08) direct antioxidants to the organelle where Fenton chemistry and mitochondrial ROS originate, whereas RNA-delivery platforms (e.g., PDA@si-UCHL3) enable genetic intervention against newly identified regulatory axes [85]. Despite these advances, long-term biosafety, tissue-residue profiles, and the mechanical durability of implanted hydrogels under cyclic loading remain unresolved and must be addressed before clinical translation.

## 7. Conclusions and Future Perspectives

Ferroptosis regulation in IVDD is, in essence, a network systematically rewired by an extreme microenvironment: avascularity plausibly creates substrate semi-starvation and an aberrant iron-source structure (with stored-iron release proposed to replace external import); mechanical overload impairs the execution enzyme through Piezo1-mediated channel signalling and downstream Ca^2+^ influx (a channel-mediated downstream effect rather than a direct action on GPX4); acidic metabolism activates ferritinophagy; and low-grade inflammation imposes a “dual lock” on SLC7A11 through NF-κB and STAT3. These mechanistic specificities constitute the value of IVDD ferroptosis research that distinguishes it from other diseases and from previous reviews.

Building on this summary, three controversies will shape the direction of subsequent research. First, is ferroptosis a cause or a consequence of IVDD? Nearly all current studies are correlational; the pivotal experiment—conditional GPX4 knockout at an early time point prior to degeneration onset—has not been performed. If ferroptosis is primarily causal, intervention should be early or even preventive; if primarily consequential, intervention should focus on halting secondary expansion of degeneration. This distinction defines the therapeutic window and should head the mechanistic research agenda.

Second, which intervention node holds the greatest promise? Head-to-head comparisons are absent. Based on the network analysis, the priority combinations to validate are “iron chelation + Nrf2 activation” and “iron chelation + NF-κB/STAT3 inhibition”—the former exerting pressure from both the “weakened attack” and “strengthened defence” sides, the latter directly releasing the “dual lock” on SLC7A11. Given that ferritinophagy is the primary iron source, the emerging NCOA4/UCHL3 targets may offer greater specificity than conventional TFR1 blockade, should the hypothesized dominance of ferritinophagy-derived iron be confirmed.

Third, cell-subtype heterogeneity has been systematically neglected. Nearly all studies use NP cells, yet the disc also contains annulus fibrosus cells, cartilaginous endplate cells, and infiltrating immune cells; ferroptotic “relay” or “bystander” effects between subtypes may exist. Single-cell RNA-seq remains unexplored in this field—potentially the next breakthrough and the greatest risk to the generalizability of current mechanistic conclusions.

Fourth, crosstalk between ferroptosis and other cell death modalities (Section 4.4) deserves equal attention. Deciphering which modality a given microenvironmental insult triggers—and whether pharmacological inhibition of one modality diverts cells toward another—will help explain the partial efficacy of single-modality interventions and argues for combined analyses of death markers in future studies.

Integrating these controversies, future research may proceed along four priority levels. (1) Pivotal gap-filling experiments: direct measurement of cystine in disc interstitial fluid, conditional GPX4-knockout time-window experiments, and direct validation of the HO-1 functional reversal under FPN1 suppression. (2) Functional dissection of new regulatory axes: in vivo validation and drug-tractability assessment of the UCHL3–HMGB1–NCOA4 and STAT3–S100A6–p53 axes. (3) Combination-strategy screening: systematic in vitro Combination Index analysis of “iron chelation + Nrf2 activation” and “iron chelation + NF-κB/STAT3 inhibition”. (4) Biomarkers: CCL3 has been reported to correlate with ferroptosis-related gene signatures in IVDD, although the evidence remains correlational and its functional role in NP cell ferroptosis is unestablished [86]—no validated specific biomarker currently exists. Reduced serum ferritin is associated with IVDD [87], suggesting that combined iron-metabolism marker panels may enable patient stratification, although circulating ferritin reflects systemic iron status rather than tissue ferritin and cannot be equated with intradiscal iron stores. Marker specificity further warrants caution because single lipid peroxidation products (e.g., MDA) can also arise from non-ferroptotic routes [88]; combinatorial panels integrating iron status (ferritin, hepcidin), lipid peroxidation products (4-HNE, MDA), and ferroptosis regulators (GPX4 activity) may ultimately prove necessary.

Two methodological advances could accelerate all four priorities simultaneously. Single-cell and spatial transcriptomics applied to human disc tissue would resolve, in one experiment, the subtype-specific ferroptosis programmes currently conflated in bulk analyses—answering which cell populations execute ferroptosis, which merely respond to it, and how ferroptotic cells signal to their neighbours. Organoid and ex vivo bioreactor models of the intervertebral disc, capable of recapitulating the coupled hypoxia–mechanics–acidity environment, would provide the missing intermediate between monolayer cultures and rodent puncture models, in which combinatorial drug screens could be performed under physiologically relevant conditions. Together, these platforms would transform the field from descriptive marker studies toward a genuinely mechanistic, testable understanding of ferroptosis in the degenerating disc.

Finally, demographic dimensions deserve attention. Estrogen regulates systemic iron metabolism, and postmenopausal women accumulate iron; whether sex-specific iron status modulates disc ferroptosis susceptibility—consistent with the sex differences in IVDD prevalence—is untested. Likewise, cellular senescence, which accumulates with age and correlates with disc degeneration, renders cells more ferroptosis-prone and directly feeds the p53-centric axes discussed in Section 4.2, suggesting that ferroptosis and senescence may mutually reinforce each other in the ageing disc.

In conclusion, the key to future breakthroughs lies not in discovering more candidate drugs but in resolving causal direction with pivotal experiments, breaking the SLC7A11 bottleneck with combination strategies, revealing subtype heterogeneity with single-cell technologies, and defining the therapeutic window with combinatorial biomarkers. Answering these questions will advance IVDD ferroptosis research from the “marker-correlation” stage to the “mechanistic-causality” stage, providing a genuinely translatable basis for targeted therapy of intervertebral disc degeneration.

## Figures and Tables

**Table 2:** Mechanistic divergence of ferroptosis between IVDD and canonical tissues.

Mechanistic Node	Canonical Tissues (Tumour/Neurodegeneration)	IVDD-Specific Mode	Mechanistic Origin of the Divergence	Evidence Strength
Function of STAT3	STAT3 → FTH1 ↑, increased iron storage, ferroptosis inhibition(“protector”) [17,18]	STAT3 → S100A6 → p53 → SLC7A11 ↓, ferroptosis promotion (non-canonical axis) [19]	Altered FTH1 promoter accessibility? Hypoxia/HIF-1α interference? (Hypothetical, awaiting validation)	Moderate (cell + animal, IVDD [19])
Primary source of labile iron	Extracellular iron import (TFR1/transferrin)	Ferritinophagy-mediated release of stored iron (NCOA4–UCHL3 axis) [20,21]	Avascular iron scarcity; acidic pH ≤ 6.5 directly activates ferritinophagy	Moderate (cell + animal, IVDD [20,21])
Mode of GPX4 inactivation	Transcriptional regulation (SLC7A11/GPX4 downregulation)	Transcriptional regulation plus mechanically induced, channel-mediated inhibition (Piezo1–Ca^2+^ axis) [22]	Sustained aberrant mechanical loading	Moderate (cell + animal, IVDD [22])
Functional property of HO-1	Net protective (antioxidant-dominant)	Potential “protective-to-deleterious” reversal when FPN1is suppressed [23,24]	IL-6–hepcidin axis blocks FPN1, preventing export of HO-1-released Fe^2+^ [25]	Hypothetical in IVDD (reversal untested); Indirect (dual-role evidence from other systems [23,24])
Cystine supply	Abundant (vascularized tissues)	Proposed endplate diffusion-limited “semi-starvation”	Avascularity + endplate calcification	Hypothetical (proposed; direct intradiscal measurements lacking)
Second defence layer (FSP1–CoQ10H_2_/GCH1–BH4/Trx)	Intact GSH-independent compensation	Fails in parallel with the GSH–GPX4 axis: NAD(P)H substrate constraints and FSP1 downregulation [26]; GCH1–BH4 and Trx unexplored	Hypoxia and nutrient deprivation are proposed to compromise the cofactors(NAD(P)H, CoQ10 precursors)that the compensatory layer requires (no direct measurements)	Moderate (human tissue omics: FSP1↓ [26]); Hypothetical (cofactor limitation proposed)
Relationship with disease	Stress event or therapeutic target	Feed-forward vicious cycle with the degenerative process [27]	Persistent low-grade inflammation + NF-κB/STAT3 crosstalk	Moderate (animal, IVDD [27])

Five mechanistic nodes diverge systematically between IVDD and canonical tissues, jointly supporting the central thesis that the extreme microenvironment rewires the ferroptosis network. Entries marked “Hypothetical” correspond to open hypotheses detailed in the main text. Arrows: ↑, upregulation/increase; ↓, downregulation/decrease; →, leads to.

**Table 3:** Pharmacological agents and strategies targeting ferroptosis in IVDD: a systematic evaluation by intervention node.

Intervention Node	Representative Agents	Key Evidence	Evidence Strength	Stage
Iron import (TFR1)	Ferristatin II [28]	TfR1 degradation → reduced iron uptake; in vivo + in vitro validation	Moderate (in vivo + in vitro, IVDD [28])	Preclinical
Labile iron chelation	DFO [29], DFOM@CeO_2_ [30]	Blocks Fenton chemistry; DFO has decades of clinical safety data in other indications	Moderate (in vivo + in vitro, IVDD [29,30])	Preclinical (clinical in other indications)
Stored-iron release (NCOA4/UCHL3) ★	PDA@si-UCHL3 [21], Pterostilbene [31], Resveratrol [26]	Targets the proposed dominant iron source in IVDD (Section 3.2); priority node recommended by this review	Moderate (in vivo + in vitro, IVDD [21,26,31])	Preclinical
Lipid peroxidation (execution end)	Fer-1 [32], Lip-1 [33,34], Quercetin [35]	Direct radical trapping independent of upstream pathway integrity	Moderate (Fer-1, Quercetin: IVDD [32,35]); Indirect (Lip-1: pancreatitis model [33,34])	Preclinical
GPX4 activity (mechanical-axis counterbalance)	Selenium supplementation (SelK) [36]	Enhances GPX4 to antagonize Piezo1-mediated ferroptosis	Moderate (cell + animal, IVDD [36])	Preclinical
Nrf2 activation (SLC7A11+)	Cynarin [37,38], Fisetin [39], Rg1 [40,41], Nystose [42], Tomatidine [43]	Upregulates GPX4/SLC7A11/HO-1; efficacy ceiling expected from the SLC7A11 “dual lock” (Section 4.3); HO-1 inducersrequire caution regarding reversal risk(Section 3.4)	Moderate (IVDD models [37,38,39,40,41,42,43]); Indirect (mechanistic branches from non-disc systems)	Preclinical
SLC7A11 (AKT1-mediated restoration)	Ethyl gallate [44]	Targets AKT1 to suppress NP cell ferroptosis and alleviate IVDD	Moderate (in vivo + in vitro, IVDD [44])	Preclinical
STAT3/NFKB1 (dual-lock release)	BRD-K20733377 [45]	The only candidate targeting both arms of the “dual lock”	Moderate (in vivo + in vitro, IVDD [45])	Preclinical
Multi-node/others	Duhuo Jisheng Decoction [46,47,48], Rg3 [49,50,51], Humanin [52,53]	Multi-target effects; Humanin from a single research group	Moderate (Duhuo Jisheng, Humanin: IVDD [46,47,48,52,53]); Indirect (Rg3: non-disc models [50,51])	Preclinical

★ Priority node recommended by this review (Section 3.2). The detailed entry-by-entry version of this table (including experimental models and outcome measures for each agent) is provided as Supplementary Table S1. Evidence levels vary by model: agents validated only in non-disc systems (Lip-1 in a pancreatitis model; Rg3 in tumour and pulmonary models; MDA as a marker in hematological systems) are cited by analogy and should not be interpreted as IVDD-specific validation.

## Data Availability

Not applicable; this is a narrative review of previously published literature.

## Supplementary Materials

The following supporting information can be downloaded at: https://www.mdpi.com/article/10.3390/biomedicines14102397/s1, Table S1: Pharmacological agents and strategies targeting ferroptosis in IVDD: detailed evaluation (21 entries).

## Abbreviations

4-HNE, 4-hydroxynonenal; AF, annulus fibrosus; AKT, protein kinase B; AMPK, AMP-activated protein kinase; BACH1, BTB and CNC homology 1; CEP, cartilaginous endplate; CoQ10, coenzyme Q10; DFO, deferoxamine; DHI, disc height index; ECM, extracellular matrix; FPN1, ferroportin-1; FSP1, ferroptosis suppressor protein 1; FTH1, ferritin heavy chain 1; GCL, glutamate-cysteine ligase; GCLC, glutamate-cysteine ligase catalytic subunit; GCLM, glutamate-cysteine ligase modulatory subunit; GPX4, glutathione peroxidase 4; GSH, glutathione; HIF-1α, hypoxia-inducible factor 1-alpha; HMGB1, high mobility group box 1; HO-1, heme oxygenase-1; IL, interleukin; IVDD, intervertebral disc degeneration; JAK, Janus kinase; LOOH, lipid hydroperoxide; LOX, lipoxygenase; MDA, malondialdehyde; MSC, mesenchymal stem cell; NCOA4, nuclear receptor coactivator 4; NF-κB, nuclear factor kappa B; NP, nucleus pulposus; Nrf2, nuclear factor erythroid 2-related factor 2; PUFA, polyunsaturated fatty acid; ROS, reactive oxygen species; SelK, selenoprotein K; SLC3A2, solute carrier family 3 member 2; SLC7A11, solute carrier family 7 member 11; STAT3, signal transducer and activator of transcription 3; TFR1/TFRC, transferrin receptor 1; TNF-α, tumour necrosis factor alpha; UCHL3, ubiquitin C-terminal hydrolase L3; ULK1, unc-51 like autophagy activating kinase 1.
