# Supplementary material for: Ferroptosis in Intervertebral Disc Degeneration: How the Extreme Microenvironment Rewires a Cell Death Network
Source: Biomedicines. 2026 Oct 20;14(10):2397. doi: 10.3390/biomedicines14102397 (PMC13702664; doi:10.3390/biomedicines14102397)
Supplement: Supplementary file 1 [file biomedicines-14-02397-s001.zip › biomedicines-4580393-supplementary.pdf]

Supplementary Table S1. Pharmacological agents and strategies targeting ferroptosis in IVDD: detailed evaluation (21 entries)

Reference numbers correspond to the main-text reference list of the manuscript.

Category	Agent	Intervention Node	Primary Target/Mechanism	Experimental Model	Key Outcome Measures	Advantages / Limitations	Research Stage	Evidence Strength	Ref
**Iron metabolism (import end)**	**Ferristatin II**	Iron import (TFR1)	Induces TfR1 internalization/degradation → reduces iron uptake; activates HIF-1α → mitophagy	Rat IVDD model; human NP tissue	TfR1↓; intracellular Fe^{2+}↓; cell viability↑	Adv: targets the iron-import entry point. Lim: single target; iron in IVDD derives mainly from stored-iron release (Section 3.2), so monotherapy efficacy may be limited	In vivo + in vitro	Moderate (rat in vivo + human NP tissue)	[28]
	**Deferoxamine (DFO)**	Labile iron chelation	Chelates labile Fe^{2+} → blocks Fenton reaction; activates HIF-1α/BNIP3 → mitophagy	Rat IVDD model; human NP cells	DHI↑; GPX4↑, ROS↓, 4-HNE↓	Adv: decades of clinical safety data. Lim: poor targeting with systemic use	In vivo + in vitro	Moderate (rat in vivo + human NP cells)	[29]
	**Deferiprone**	Labile iron chelation	Same as DFO — chelates labile iron and promotes excretion	No IVDD data	N/A	Adv: high oral bioavailability. Lim: unvalidated in IVDD	Clinical in other indications	Not applicable (no IVDD data)	—
	**DFOM@CeO_2 nanoparticles**	Labile iron chelation + GPX4 enhancement	DFOM chelates iron + CeO_2 enhances GPX4 activity and degrades lipid peroxides; activates mitochondrial DHODH-$CoQH_2$ pathway	Rat IVDD model; human NP cells	DHI↑; GPX4↑, 4-HNE↓; mitochondrial ROS↓	Adv: local sustained release, multi-target. Lim: complex synthesis; long-term biosafety unverified	In vivo + in vitro	Moderate (rat in vivo + human NP cells)	[30]
**Iron metabolism (stored-iron release) ★**	**PDA@si-UCHL3 nanoplatform**	Ferritinophagy (UCHL3)	Silences UCHL3 → blocks HMGB1 stabilization → suppresses NCOA4-mediated ferritinophagy; also scavenges ROS and chelates iron (tri-functional)	Rat IVDD model; human NP cells	Ferritinophagy↓; ferroptosis↓; degeneration alleviated	Adv: directlytargets the proposed dominant iron source (Section 3.2); multi-functional. Lim: RNAi delivery efficiency, off-target risk	In vivo + in vitro	Moderate (rat in vivo + human NP cells)	[21]
	**Pterostilbene**	Ferritinophagy (NCOA4)	Inhibits ferritinophagy-mediated ferroptosis via the NRF2/NCOA4/FTH1 axis; nanoliposomal delivery	Rat IVDD model	NCOA4↓, FTH1↑; ferroptosis↓	Adv: targets the NCOA4 axis (priority node). Lim: relies on nanoformulation	In vivo	Moderate (rat in vivo)	[31]
	**Resveratrol**	Ferritinophagy (NCOA4) + lipid peroxidation	Multi-omics show NCOA4↓ and GPX4/FSP1↑; phenolic hydroxyl terminates lipid peroxidation; anti-inflammatory	Rat IVDD model; human NP cells	NCOA4↓; DHI↑; IL-1β/TNF-α/IL-6↓	Adv: dual-node action (ferritinophagy + lipid peroxidation). Lim: very low oral bioavailability	In vivo + in vitro	Moderate (rat in vivo + human NP cells)	[26]
**Lipid peroxidation inhibitors**	**Ferrostatin-1 (Fer-1)**	Lipid peroxidation (execution end)	Arylamine structure traps lipid peroxyl radicals → terminates chain reaction	Rat IVDD model; human NP cells	Cell viability↑; GSH restored; ROS↓, MDA↓	Adv: gold-standard tool compound; upstream-pathway-independent. Lim: short half-life, poor solubility	In vivo + in vitro	Moderate (rat in vivo + human NP cells)	[32]
	**Liproxstatin-1 (Lip-1)**	Lipid peroxidation (execution end)	Same as Fer-1, higher potency	Validated in pancreatitis model; limited IVDD data	Same as Fer-1	Adv: more stable. Lim: IVDD-specific data limited (cited by analogy)	In vivo (other organs) + in vitro	Indirect (pancreatitis model; no IVDD data)	[33,34]
	**Quercetin**	Lipid peroxidation + iron chelation	Phenolic hydroxyl interrupts lipid peroxidation; inhibits LOX/COX; chelates metal ions (OPN-mediated ferroptosis in hydrogel form)	Rat IVDD model (nanoformulation-embedded hydrogel); human NP cells	ROS↓; lipid peroxides↓; ferroptosis↓	Adv: natural multi-target. Lim: formulation-dependent delivery	In vivo + in vitro	Moderate (rat in vivo + human NP cells)	[35]
	**Selenium supplementation (SelK pathway)**	GPX4 activity (mechanical-axis counterbalance)	Upregulates SelK → enhances GPX4 activity → antagonizes Piezo1-mediated ferroptosis	Mechanical overloading model; NP cells	GPX4 activity↑; oxidative stress↓; disc senescence attenuated	Adv: the only strategy countering the mechanical axis (Section 2.2); nutritional-grade safety. Lim: dosing window undefined	In vivo + in vitro	Moderate (mechanical overloading model, NP cells)	[36]
**Cell defense & signaling modulators**	**Cynarin**	Nrf2 activation (SLC7A11+)	Binds Keap1 → Nrf2 nuclear translocation → upregulates GPX4/SLC7A11/HO-1/FTH1; suppresses NF-κB	Rat IVDD model; human NP cells	GPX4↑, SLC7A11↑; Fe^{2+}↓; cytokines↓	Adv: complete evidence chain. Lim: Nrf2-only activation faces the "dual lock" ceiling (Section 4.3)	In vivo + in vitro	Moderate (rat in vivo + human NP cells)	[37,38]
	**Fisetin**	Nrf2 activation + AMPK	Activates Nrf2 and AMPK; suppresses JAK2/STAT3; delays senescence	Rat IVDD model; human NP cells	Nrf2↑, GPX4↑; p16/p53↓	Adv: triple effect (anti-ferroptosis + anti-senescence + anti-inflammatory). Lim: human PK data lacking	In vivo + in vitro	Moderate (rat in vivo + human NP cells)	[39]
	**Ginsenoside Rg1**	Nrf2 activation	Binds Nrf2/GPX4/SLC7A11/FTL; miR-146a-5p/SIRT1 axis enhances Nrf2 stability	Rat IVDD model; multi-omics	ROS↓, MDA↓; ML385 reverses the effects	Adv: multi-omics-validated targets. Lim: same single-axis ceiling	In vivo + in vitro	Moderate (rat in vivo + multi-omics)	[40,41]
	**Nystose (fructo-oligosaccharide)**	Nrf2 activation	Directly binds Nrf2 → promotes nuclear translocation → activates Nrf2/HO-1/GPX4 axis	Human NP cells; Nrf2-silencing model	ROS↓; ferroptosis↓; ECM homeostasis restored	Adv: Nrf2 silencing abolishes protection (target specificity). Lim: limited in vivo IVDD data	In vitro (IVDD); in vivo in other systems	Moderate (human NP cells in vitro)	[42]
	**Tomatidine**	Nrf2/HO-1 (caution: reversal risk)	Activates Nrf2/HO-1/GPX4 pathway	Rat IVDD model	Ferroptosis↓; degeneration alleviated	Adv: natural product. Lim: HO-1 induction under FPN1 suppression may reverse to pro-death (Section 3.4); timing critical	In vivo	Moderate (rat in vivo)	[43]
	**Ethyl gallate**	SLC7A11 (AKT1-mediated restoration)	Targets AKT1 → suppresses ferroptosis	Rat IVDD model	Ferroptosis↓; degeneration alleviated	Adv: targets AKT1, upstream of the SLC7A11–GPX4 defense. Lim: long-term effects unevaluated	In vivo + in vitro	Moderate (rat in vivo)	[44]
	**Duhuo Jisheng Decoction (DHJSD)**	Multi-node	Contains gallic acid-like components; exosomal miR-19b-3p → ACSL4↓; activates PI3K/AKT	Rat IVDD model (oral gavage)	Fe^{2+}↓, ROS↓; ACSL4↓	Adv: multi-target formula. Lim: complex components, batch QC challenges	In vivo	Moderate (rat in vivo)	[46–48]

	**Ginsenoside Rg3**	AMPK	Targets PRKAA2 (AMPKα2 subunit) → activates AMPK	Rat IVDD model; network pharmacology	Cell viability↑; disc height maintained	Adv: mechanistically distinct from Rg1; cross-disease generality (tumor/pulmonary models, cited by analogy). Lim: combination effects unexplored	In vivo + in vitro	Moderate (rat IVDD model); Indirect (tumor/pulmonary models, cited by analogy)	[49–51]
	**BRD-K20733377**	STAT3/NFKB1 (dual-lock release)	Targets the STAT3/NFKB1 axis → upregulates GPX4, FTH1, SLC7A11	Rat IVDD model; NP-MSCs; AI-driven screening	Fe^{2+}↓; GPX4↑; pain behavioral scores improved	Adv: only candidate hitting both arms of the "dual lock" (Section 4.3). Lim: metabolic pathway incompletely characterized	In vivo + in vitro	Moderate (rat in vivo + NP-MSCs)	[45]
**Endogenous signaling peptides**	**Humanin (HN)**	Mitochondria + STAT3/NF-κB	Upregulates HSP27 → suppresses JAK2/STAT3/NF-κB; maintains mitochondrial homeostasis	Rat IVDD model; human NP cells	STAT3 phosphorylation↓; mtROS↓; GPX4↑	Adv: low immunogenicity. Lim: single research group; peptide delivery barriers	In vivo + in vitro	Moderate (rat in vivo + human NP cells)	[52,53]

★ Priority node recommended by this review (Section 3.2).Each agent is graded in the Evidence Strength column according to the four-level scheme defined in Section 1.4 of the manuscript (Strong / Moderate / Indirect / Hypothetical); agents validated only in non-disc systems (Lip-1 in a pancreatitis model; Rg3 in tumor and pulmonary models) are labelled Indirect, cited by analogy, and should not be interpreted as IVDD-specific validation.Stage color code: green = clinical (other indications); blue = in vivo animal validation; red = in vitro only. Abbreviations: DHI, disc height index; ECM, extracellular matrix; NP, nucleus pulposus; BMSC, bone marrow mesenchymal stem cell; mtROS, mitochondrial ROS.
